# Editorial: Innovation in developmental psychology, education, sports, and arts: advances in research on individuals and groups

**DOI:** 10.3389/fpsyg.2023.1260109

**Published:** 2023-10-03

**Authors:** Georgeta Pânişoara˘, Radu Predoiu, Alexandra Predoiu, Andrzej Piotrowski

**Affiliations:** ^1^Faculty of Psychology and Educational Sciences, University of Bucharest, Bucharest, Romania; ^2^Faculty of Physical Education and Sport, Teachers' Training Department, National University of Physical Education and Sports, Bucharest, Romania; ^3^Faculty of Physical Education and Sport, Sports and Motor Performance Department, National University of Physical Education and Sports, Bucharest, Romania; ^4^Institute of Psychology, University of Gdańsk, Gdańsk, Poland

**Keywords:** performance improvement, developmental growth through sports, interplay, forming skills in children, teenagers and adults

This Research Topic seeks to generate relevant information for psychologists, educators, and sports science specialists to facilitate growth and development in children, teenagers, and adults, increasing their performance levels.

Due to the ever-expanding wealth of knowledge surrounding us, we find ourselves constantly “besieged” by a multitude of data that entice our attention. Children's, adolescents', and adults' social, emotional, intellectual, and psychomotor development needs to be taken into account when determining their ability to meet the demands of education or a given sport or arts.

Growth and technological advances in the areas of educational psychology, sport, and arts have changed considerably over time, in relation to students' and athletes' preparation and performance. Technology supports specialists, with chatbot technology (an artificial intelligence—AI application) being used to improve learning practices and teaching (Okonkwo and Ade-Ibijola, 2021). The results of the study developed by Al-Abdullatif et al. showed that the use of AI-based tools, such as chatbots, enhances students' learning strategies and learning motivation. More exactly, the students who used the chatbot system showed more favorable levels of cognitive and metacognitive learning strategies and, also, demonstrated a higher level of self-efficacy for learning and higher scores in the case of perceived task value.

The manuscripts in this Research Topic explore the interplay between individual and environment: (1) how the COVID-19 pandemic affected university students in Turkey (Aslan and Çinar); (2) if birthplace (and relative age effect) is linked to athletes' sport performances (Thuany, Vieira et al.); (3) the influence of parenting style on children's outcomes (Şiţoiu and Pânişoară); (4) how the conditions in which preteens live (preteens from single-parent families, students having a parent working abroad, socially assisted students, and Roma preadolescents) influence their temperamental characteristics and behavior (Moanţă et al.); (5) how caregivers of individuals with Down syndrome can change their perspective on their own life to handle adversities and challenging situations (Chiracu et al.); (6) the effects (on students' motivation and learning strategies) of implementation of a chatbot in Saudi higher education (Al-Abdullatif et al.); (7) the subject–environment interplay among runners from different Brazilian macro-regions (Thuany, Bandeira et al.); (8) the effectiveness of a dual career support system on Swedish athletes performances (Nyberg et al.); and (9) how risk-taking behavior and trait anxiety influence athletes' injury severity in competitions (Patenteu et al.).

Interpreted by many as a eulogy to physical exercise, Juvenal's dictum—“Mens sana in corpore sano”—cultivates the spirit of equal status, and even, according to the cliché, primacy. In an increasingly technological world, the concern of children (and parents), adolescents, and adults for their own growth and development is essential for their wellbeing, health, and productivity in the decades to come, and in turn, for their nations and communities to thrive. The study by Şiţoiu and Pânişoară recommends the development of emotional intelligence (associated with the adoption of an authoritative parenting style) and self-esteem in parents. The two variables determine parental competencies (authors found that self-esteem plays a mediating role in the relationship between parents' emotional intelligence and parenting competence). The training of adults in terms of parental education is essential, the Barnum effect being felt regardless of parents' status or educational level.

With the suspension of traditional school education (during the COVID-19 pandemic), the difficulties for teaching became more serious, “due to the change in teacher–student relationships, but especially at early ages” (Quílez-Robres et al., 2022). The temperamental features of preteens at risk of educational and social exclusion (from disadvantaged classes—rural areas and those having low socioeconomic levels) were examined by Moanţă et al.. The authors stressed the need for specialists to decrease negative affectivity (including fear and frustration) and depressive mood in preadolescents and to increase effortful control—including inhibitory control and attention. Moreover, they asserted the need for a temperament-conscious education in parental education and teacher training.

However, the changes brought by the COVID-19 pandemic are experienced differently according to the person's system of reference—“the same factors might be perceived as barriers […] or as opportunities for growth and development” (Drugas et al., 2023). The research of Aslan and Çinar revealed that during the COVID-19 pandemic, special attention must be paid to religious level, job loss, deterioration of economic status, gender, relationship status, and physical activities, as these variables have the potential to increase distress, anxiety, and depression level in university students.

We also found research, such as the one by Chiracu et al., which emphasizes that having a strong psychological capital (PsyCap), including hope, self-efficacy, optimism, and resilience, enables the caregivers of individuals with Down syndrome to report higher levels of perceived quality of life. PsyCap represents an inner resource of caregivers to handle adversities and challenging situations and to perceive a higher level of subjective wellbeing.

Psychology often makes the difference between the first places and the other positions in the ranking. On the other hand, variables such as relative age effect (RAE) and birthplace (various regions present economic and social differences, influencing training facilities, and sports practice) can also make the difference when talking about athletes' sports performances. A higher frequency of Olympic athletes from Brazil was born in the first and second quartiles, while most of them were born in the Southeast region of the country, especially São Paulo state (Thuany, Vieira et al.). Exploring the educational background of Swedish world-class athletes, Nyberg et al. answered the question of whether different forms of Dual Career Support (DCS) have the potential to help talented athletes reach international-level performances. It seems that taking part in DCS is not essential for young athletes to register success (only 44% of world-class athletes had taken part in some form of DCS). Areas of science in which “practice lag behind research evidence are known as *valleys of death”* (Evans and Brewer, 2022). Therefore, it is required to advance the application of psychology in the sports science field, and, also, with respect to sports injury prevention and rehabilitation. According to Patenteu et al., a higher level of instrumental risk and a moderate or slightly below-average level of anxiety in unusual, new circumstances are linked with a decreased likelihood of severe injuries in athletes (competitive martial arts athletes were investigated). Moreover, in the case of Mixed martial arts (MMA), a higher value for anxiety in physically dangerous conditions is associated with more severe injuries.

Not least, environmental indicators, such as public illumination, sidewalks, green areas, perception about the weather, and perception about the built environment or pavement, were examined, as key factors influencing training and practice commitment among runners from different Brazilian macro-regions (Thuany, Bandeira et al.). Network topologies are different, and runners' performance could also be context-driven. On the other hand, it is known that the effect of deliberate practice on performance is larger for highly predictable activities (e.g., running) than for less predictable ones (e.g., handling a medical emergency) (Macnamara et al., 2014).

Educational psychology and psychological growth through sports are all in expansion. The extent to which the two environments spur growth is giving them value. In this Research Topic, there are relevant contributions that help specialists (teachers, psychologists, social workers, coaches, etc.) work with children, adolescents, or adults, guiding them to pay constant attention to progressive development and the ability to learn, change, and adapt.

## Author contributions

GP: Writing—original draft. RP: Writing—original draft. AP: Writing—review and editing. AP: Writing—review and editing.

## References

[B1] Drugas M. I. Roncaglia I. Rothmann S. Stoyanova S. Y. (2023). Editorial: Well-being and work motivation brought by technological changes, coping, and adaptations during and post COVID-19 pandemic: barriers and opportunities. Front. Psychol. 14, 1150726. 10.3389/fpsyg.2023.1150726 36874831 PMC9978707

[B2] Evans L. Brewer B. W. (2022). Applied psychology of sport injury: getting to–and moving across–the valley of death. J. Appl. Sport Psychol. 34, 1011–1028. 10.1080/10413200.2021.2015480

[B3] Macnamara B. N. Hambrick D. Z. Oswald F. L. (2014). Deliberate practice and performance in music, games, sports, professions, and education: a meta-analysis. Psychol. Sci. 25, 1608–1618. 10.1177/0956797614535810 24986855

[B4] Okonkwo C. W. Ade-Ibijola A. (2021). Chatbots applications in education: a systematic review. Comput. Educ. Artif. Intell. 2, 100033. 10.1016/j.caeai.2021.100033

[B5] Quílez-Robres A. Latorre-Cosculluela C. Moyano N. (2022). Editorial: educational experiences for the development of the SDG in the time of COVID-19. Front. Educ. 7, 927391. 10.3389/feduc.2022.927391

